# Correction: Preisegger et al. Visualizing Oral Infection Dynamics of *Beauveria bassiana* in the Gut of *Tribolium castaneum*. *J. Fungi* 2025, *11*, 101

**DOI:** 10.3390/jof11060409

**Published:** 2025-05-26

**Authors:** Lautaro Preisegger, Juan Cruz Flecha, Fiorella Ghilini, Daysi Espin-Sánchez, Eduardo Prieto, Héctor Oberti, Eduardo Abreo, Carla Huarte-Bonnet, Nicolás Pedrini, Maria Constanza Mannino

**Affiliations:** 1 Instituto de Investigaciones Bioquímicas de La Plata (INIBIOLP), CCT La Plata Consejo Nacional de Investigaciones Científicas y Técnicas (CONICET), Universidad Nacional de La Plata (UNLP), Calles 60 y 120, La Plata 1900, Argentina; lpreisegger@med.unlp.edu.ar (L.P.); juancruzflecha16@gmail.com (J.C.F.); despinsanchez@med.unlp.edu.ar (D.E.-S.); carlahb@biol.unlp.edu.ar (C.H.-B.); 2 Instituto de Investigaciones Fisicoquìmicas Teóricas y Aplicadas (INIFTA), CCT La Plata Consejo Nacional de Investigaciones Científicas y Técnicas (CONICET), Universidad Nacional de La Plata (UNLP), Diagonal 113 y 64 S/N, La Plata 1900, Argentina; fiorelag@inifta.unlp.edu.ar (F.G.); edprieto@quimica.unlp.edu.ar (E.P.); 3 Instituto de Ciencias de la Salud, Universidad Nacional Arturo Jauretche (ICS-UNAJ), Avenida Calchaqui, Florencio Varela 6200, Argentina; 4 Laboratorio de Bioproducción, Plataforma de Bioinsumos, Instituto Nacional de Investigación Agropecuaria, Estación Experimental Wilson Ferreira Aldunate, Ruta 48 Km 10, Canelones 90000, Uruguay; hoberti@inia.org.uy (H.O.); eabreo@inia.org.uy (E.A.); 5 Departamento de Biotecnología, Universidad ORT Uruguay, Montevideo 10129, Uruguay

In the original article [1], the omission of two critical references led to a shift in the citation numbering throughout the discussion section, which significantly impacts the interpretation of the text. The specific changes and their implications are detailed below:

**Adding References 27 and 28**: These references were omitted in the editing process, they were in the original manuscript and when it was sent to review if it was correct, they were missing from the list, hence the reference numbering was wrong. Now, they are all included and the numbering should be 27. Kilaru; 28. Bernasconi and 29. Oberti, since that is the correct referencing according to what is written in the main text in the manuscript.

**Reference 27:** Kilaru, S.; Schuster, M.; Studholme, D.; Soanes, D.; Lin, C.; Talbot, N.J.; Steinberg, G. A codon-optimized green fluorescent protein for live cell imaging in Zymoseptoria tritici. *Fungal Genet Biol*. **2015**, *79*, 125–131. https://doi.org/10.1016/j.fgb.2015.03.022.

**Reference 28:** Bernasconi, A.; Alassimone, J.; McDonald, B.A.; Sánchez-Vallet, A. Asexual reproductive potential trumps virulence as a predictor of competitive ability in mixed infections. *Environ. Microbiol.* **2022**, *24*, 4369–4381. https://doi.org/10.1111/1462-2920.16018.

Volume and page was added in reference 29 as one of the sources was a preprint and has since been published.

**Reference 29:** Oberti, H.; Sessa, L.; Oliveira-Rizzo, C.; Di Paolo, A.; Sánchez-Vallet, A.; Seidl, M.F.; Abreo, E. Novel genomic features in entomopathogenic fungus *Beauveria bassiana ILB308*: Accessory genomic regions and putative virulence genes involved in the infection process of soybean pest Piezodorus guildinii. *Pest. Manag. Sci.* **2025**, *81*, 2323–2336. https://doi.org/10.1002/ps.8631.

Reference 27 should be Kilaru as is shown in the updated version. Oberti should be reference 29, since the techniques cited were published in Kilaru (Ref. 27) and Bernasconi (Ref. 28) and in Oberti et al., 2025 (Ref. 29).

These references provide essential background and methodology details that are foundational to the discussion of the results. Their omission not only diminishes the scientific rigor of the article but also disrupts the logical flow of the argument presented.

The omission has caused all subsequent citations to be misnumbered, starting from Section 2.1.1. This misnumbering altered the meaning of several key points in the discussion, as the numerical citations pointed to incorrect sources, potentially misleading readers.

The authors state that the scientific conclusions are unaffected. This correction was approved by the Academic Editor. The original publication has also been updated.
