# Supplementary material for: Visualizing Oral Infection Dynamics of Beauveria bassiana in the Gut of Tribolium castaneum
Source: J Fungi (Basel). 2025 Jan 28;11(2):101. doi: 10.3390/jof11020101 (PMC11856336; doi:10.3390/jof11020101)

Supplementary information

Visualizing oral infection dynamics of *Beauveria bassiana* in the gut of *Tribolium castaneum*

Lautaro Preisegger; Juan Cruz Flecha; Fiorella Ghilini; Daysi Espin-Sánchez; Eduardo Prieto; Héctor Oberti; Eduardo Abreo; Carla Huarte-Bonnet; Nicolás Pedrini; Maria Constanza Mannino

Figure S1. Characterization of alginate capsules: Images of both control (A, B) and conidia-containing capsules (C, D) in their hydrated state immediately after preparation (A, C) and after 72 hours of drying at 26°C (B, D). Diameter (mm) of capsules in their hydrated state (E) and after drying (F). Thermal (G) and oxidative stress (H) tolerance assays showing sporulation for each treatment after 10 days at 26ºC. In (G) and (H), the light grey bars correspond to conidia powder used as a control, and the dark grey bars correspond to encapsulated conidia. Bars represent mean ± SD (N=3). Statistical significance was determined using Student's *t*-test (NS: not significant; *: $p < 0.05$; **: $p < 0.01$).

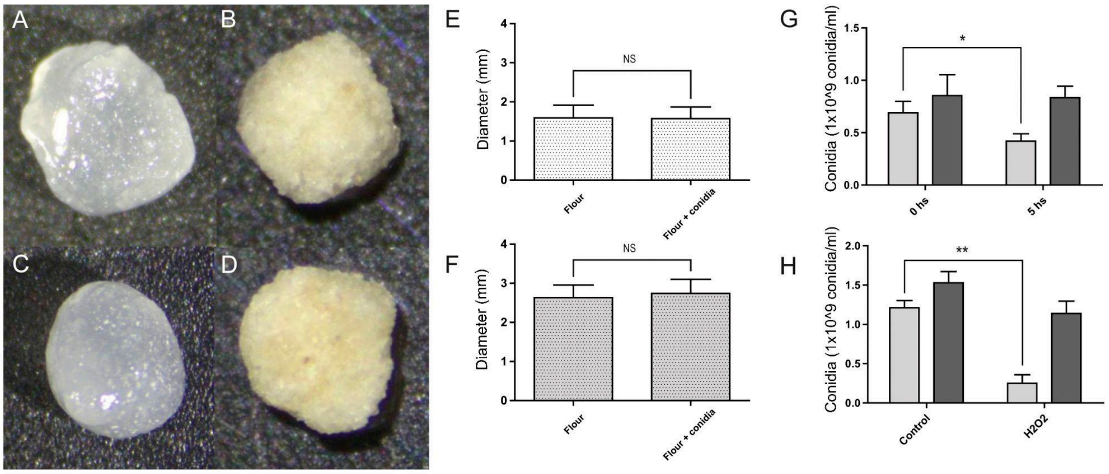

Figure S2. Atomic Force Microscopy (AFM) measurements: Capsule topology was characterized by measuring amplitude error, height and phase for control (A-C) and conidia-containing (D-F) capsules. A 5 μm area was analyzed.

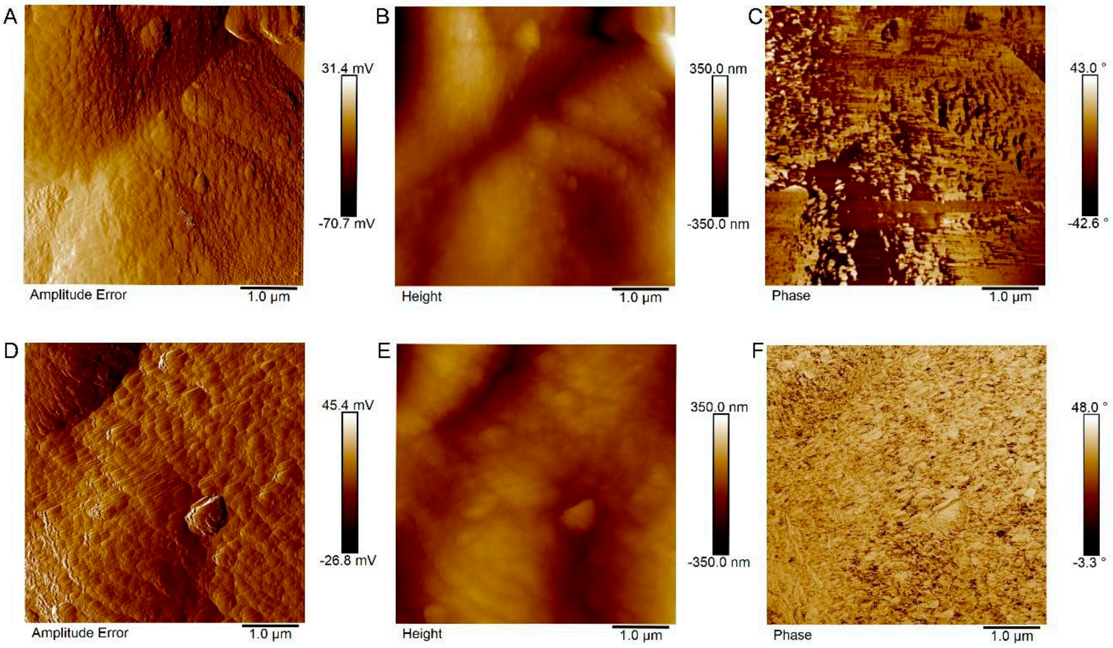
A
31.4 mV
-70.7 mV
Amplitude Error
1.0 µm
B
350.0 nm
-350.0 nm
Height
1.0 µm
C
43.0 °
-42.6 °
Phase
1.0 µm
D
45.4 mV
-26.8 mV
Amplitude Error
1.0 µm
E
350.0 nm
-350.0 nm
Height
1.0 µm
F
48.0 °
-3.3 °
Phase
1.0 µm

Figure S3. Confocal microscopy characterization of *B. bassiana* and *T. castaneum*: *B. bassiana* ARSEF 2860 EGFP conidia suspension (A-C). The red quadrant shown in (A) is enlarged in (E-G). *T. castaneum* PyC autofluorescence (I-K). Intensity quantification for red (red line) and green (green line) signals shown in (D, H, L). Red arrows indicate conidia groups. Channels are indicated in the top right of the top panels. Yellow arrows mark the horizontal line where signal quantification was performed.

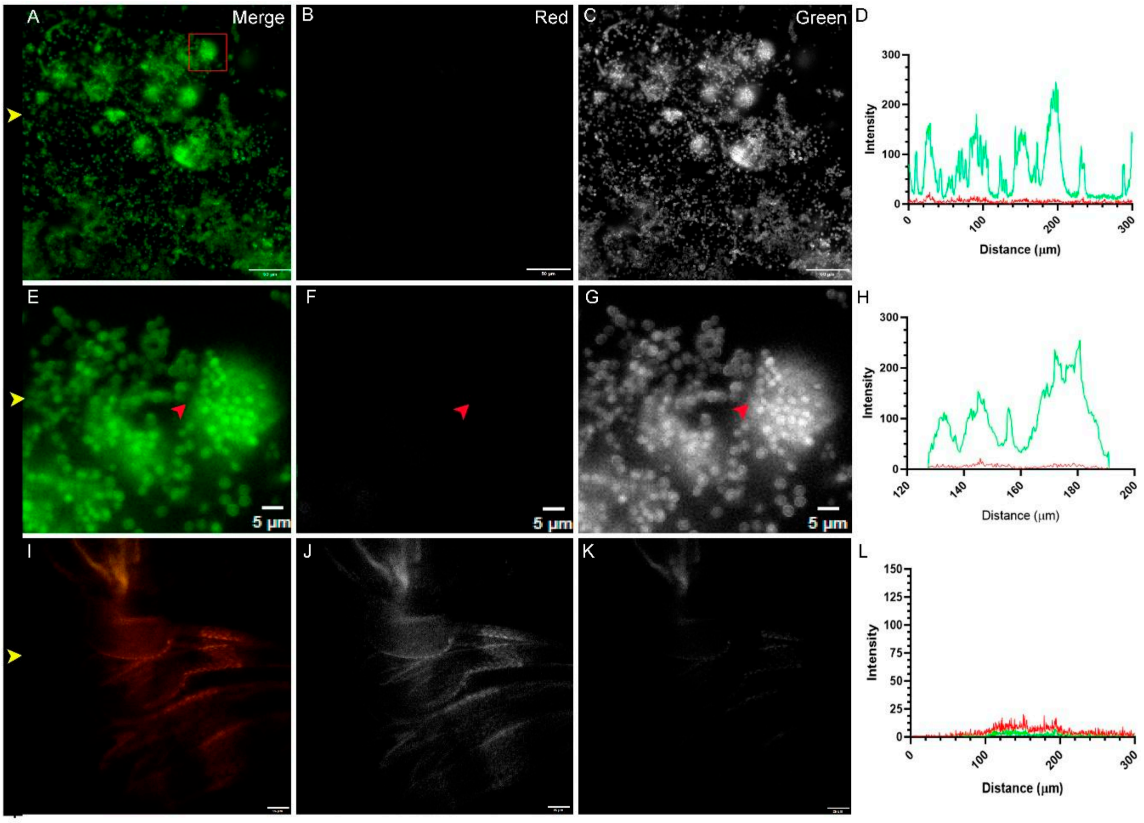

Figure S4. Conidia quantification in the alimentary canal after feeding with encapsulated formulation. (A) *B. bassiana* colony-forming units (CFUs) from the alimentary canals of *T. castaneum* larvae and adult individuals. Adult and larval *T. castaneum* were surface-washed and dissected after exposure to *B. bassiana* conidia. Colonies were counted in each replicate, and concentrations were calculated as conidia/ml. (B) Conidia count from confocal images of the hindgut and midgut of larvae and adults. Conidia were counted in quadrants, and quadrant area was used to normalize counts and define arbitrary units (AU). Statistical significance was determined using a Student's *t*-test (***: $p < 0.001$, ****: $p < 0.0001$).

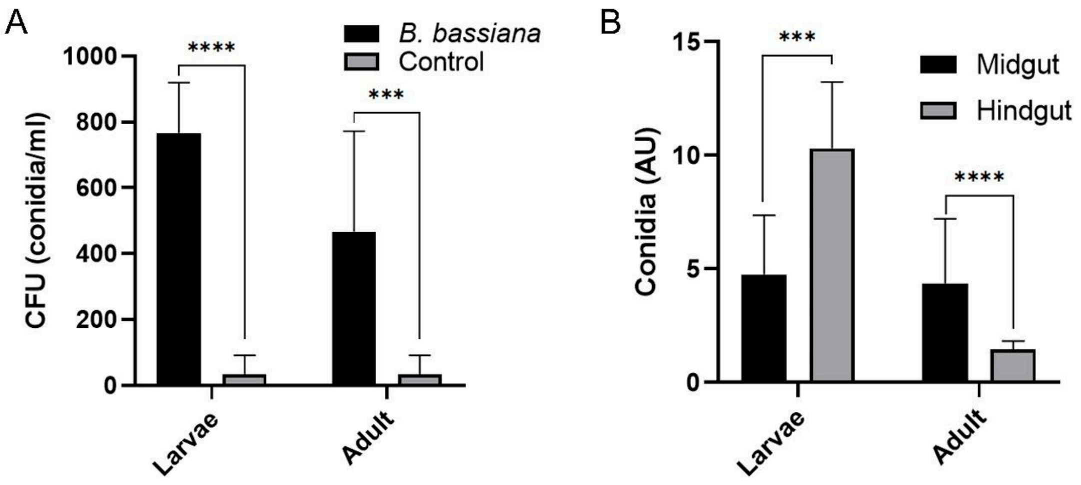

Supplement: Supplementary file 1 [file jof-11-00101-s001.zip › jof-3434306-supplementary.pdf]
